# Geospatial distribution of relative cesarean section rates within the USA

**DOI:** 10.1186/s13104-022-06141-w

**Published:** 2022-07-15

**Authors:** Rahul S. Yerrabelli, Nicholas Peterman, Bradley Kaptur, Eunhae Yeo, Kristine Carpenter

**Affiliations:** 1 Carle Illinois College of Medicine, University of Illinois at Urbana-Champaign, Champaign, llinois 61820 USA; 2 Carle Foundation Hospital, Urbana, IL 61801 USA

**Keywords:** Cesarean section, Obstetrics, USA, Demographics, Geography, Rural, RUCA, Geospatial analysis, Moran’s I, Spatial autocorrelation

## Abstract

**Objective:**

To evaluate the existence of statistically significant clusters of Cesarean section rates at the county level and assess the relationship of such clusters with previously implicated socioeconomic factors.

**Results:**

County-level obstetrics data was extracted from March of Dimes, originally sourced from National Center for Health Statistics. County-level demographic data were extracted from the US Census Bureau. Access to obstetricians was extracted from National Provider Identifier records. Rural counties were identified using Rural Urban Commuting Area codes developed by the department of agriculture. The dataset was geospatially analyzed using Moran’s I statistic, a metric of local spatial autocorrelation, to identify clusters of increased or decreased Cesarean section rates. The American South, especially the Deep South, is a major cluster of increased Cesarean section rates. As a general but not absolute pattern, the American West and Midwest had lower Cesarean section rates than the Northeast. Focal areas of increased Cesarean section rates included the Kansas-Nebraska border, Michigan’s upper peninsula, and the New York City metropolitan area. The gross geospatial differences were not explained by rurality, obstetric access, or ethnic and racial factors alone.

**Supplementary Information:**

The online version contains supplementary material available at 10.1186/s13104-022-06141-w.

## Introduction

When medically justified, the Cesarean section (C-section) is a life-saving intervention that greatly reduces maternal and perinatal morbidity and mortality. However, C-sections are surgical interventions and, without a medical indication, confer all the risks of surgery and anesthesia including longer recovery times and hospital stays, greater pain levels, and increased risks for later vaginal deliveries and obstetric or non-obstetric abdominal surgeries (due to adhesions). Rising Cesarean section rates over the past few decades have raised significant concern, although the causes are multifactorial and difficult to elucidate. Causes could include risk avoidance with regards to malpractice concerns, overuse or overreliance of new diagnostic testing, patient misconceptions, or convenience to medical staff.

A so-called “ideal” C-section rate has been a significant matter of controversy. Since a 1985 WHO analysis [1, 2] proposed a ceiling of 10–15%, this number has been often cited in the literature and used in practice. While more modern meta-analyses [3, 4] have supported the notion that higher C-section rates do not confer any additional benefit to maternal or perinatal outcomes, there are significant limitations with this target—or any fixed target—including the lack of randomized controlled data, disregard for the complexity of a population, disregard for the type of C-section (repeat vs primary) or the indication, and that the rate was originally proposed for population levels (as opposed to hospital systems, which is the usual category upon which the number is applied). Regardless, the C-section rate in the United States of America (USA) has been above 20% since at least the 1990s and is now approximately 30–32% as of 2019 [5]. While the USA does outperform some nations worldwide (Brazil, Dominican Republic, Greece, Cyprus, Egypt, Turkey all have > 50% C-section rates), the USA does have higher rates than most developed nations [6]. And while the C-section rate has increased in the last three decades, so have maternal mortality and morbidity, which has disproportionately impacted women of color compared to women of other races [5, 7].

Previous works have assessed the access and evolution of Cesarean section utilization in the United States [8–10]. The purpose of this work is to use geospatial analysis techniques to identify the existence of statistically significant utilization clusters and evaluate the relationship of previously implicated socioeconomic variables at a county level between cluster categorizations. This work aims to expand on previous works by (1) using county-level socioeconomic variables to evaluate the association of local environment and maternal and perinatal health, (2) using cluster designations to identify hotspots for targeted intervention efforts, and (3) using a subset of cluster designations (i.e., High-Low and Low–High) to evaluate these variables in counties that uniquely differ from the surrounding counties.

## Main text

### Data sources, collection, and extraction

This study utilized multiple publicly available datasets from the March of Dimes, United States Census Bureau, and National Plan and Provider Enumeration System (NPPES). All data were acquired on a per-county level and year-matched for 2016–2019. Python was utilized for database building and GeoDa was used for visualization and analysis.

The core pregnancy dataset was derived from the March of Dimes county-level metrics and included the percent of live births that utilized a Cesarean section, that were preterm (< 37 weeks gestational age), and that were low-birthweight (< 2500 g) [11]. This March of Dimes data was originally sourced from the National Center for Health Statistics [12]. Race-stratified pregnancy metrics were not available at a county level. To obtain the birth rate as well as the socioeconomic attributes of each county in the chosen time range, American Community Survey data from the US Census Bureau was utilized [13]. Select county-level socioeconomic metrics included percent in poverty, median household income, Rural Urban Commuting Area (RUCA) codes, insurance coverage, population density, racial demographics, education level, and family size. Because of this paper’s focus on the community level access and prevalence of Cesarean sections, community-level racial demographics, rather than the racial metrics of the mothers themselves, were determined to be the core racial metric of the overall analysis. RUCA codes were developed by the US Department of Agriculture and rank each county on an urbanization scale from 1 to 9, with 9 being the most rural [14].

NPPES National Provider Identifier (NPI) records, containing the specialty and practice location of all physicians in the USA, were then filtered by specialty and used to determine the number of obstetricians actively practicing in each county [15].

Three counties were filtered out for incomplete or missing datasets in any of the aforementioned variables with 3105 counties of 3108 mainland USA counties remaining in the analysis. The remaining dataset was exported to GeoDa, a geospatial analysis software, for analysis and visualization [16]. Core demographic, socioeconomic, and pregnancy variables were visualized in county-level map form.

### Statistical analyses

A Moran’s I cluster analysis was performed to determine statistically significant, p ≤ 0.05, spatial variations in Cesarean section prevalence. Moran’s I statistic is a measure of spatial correlation commonly used in geography. For a variable of interest, the Moran’s I statistic examines both a county’s value and a county’s average neighbor’s value and describes their relationship to the national average value. If both a county and its neighbors have statistically significantly higher or lower Cesarean prevalence than the national average, then that county is considered to be statistically significant as a whole and further classified as either High-High, Low-Low, Low–High, or High-Low. The first High or Low attribute represents the county’s value in comparison to the nation. The second High or Low attribute represents the county’s neighbor’s value in comparison to the nation. High-High and Low-Low groups can be conceptualized as hotspots and coldspots respectively. Low–High and High-Low groupings represent geospatial outliers and mark areas of incongruence on a map- these may be areas that are different only because of reflexive compensation around a coldspot or hotspot. Moran’s I cluster analysis was similarly performed on racial, obstetrician, and urbanization metrics.

The criteria for determining whether one county is considered a neighbor of another is based on the distance between the county centroids. A threshold of approximately of 150 km (approximately 90 miles) was used as it was calculated as the minimum nearest neighbor distance to allow for each county to have at least one neighbor, which is a requisite of further cluster analysis.

The four significant Moran’s I groupings of Cesarean section prevalence were then placed in a one-way ANOVA across all county-level socioeconomic and pregnancy variables to identify statistically significant disparities. A two-tailed T-test was similarly conducted across the same variables comparing only High-High (hotspot) and Low-Low (coldspot) groupings.

Unless otherwise stated, a significance threshold of p ≤ 0.05 was used.

## Results and discussion

### National level statistics

10,535,494 births were identified in the mainland USA over the time period of interest between 2016 and 2019. Of these births, 9.93% were preterm, 8.16% were low-birthweight, and 31.78% were conducted with a Cesarean Sect. 72.75% of counties contained at least 1 obstetrics-gynecology physician, with a total of 58,271 obstetrics-gynecology physicians included in the analysis overall. The average percent of families living in poverty was 8.16% with 30.90% of single mother families living in poverty and 33.74% of births to unmarried couples. 5.46% of households contained a single parent.

### Overall geographic clustering

**Fig. 1:**
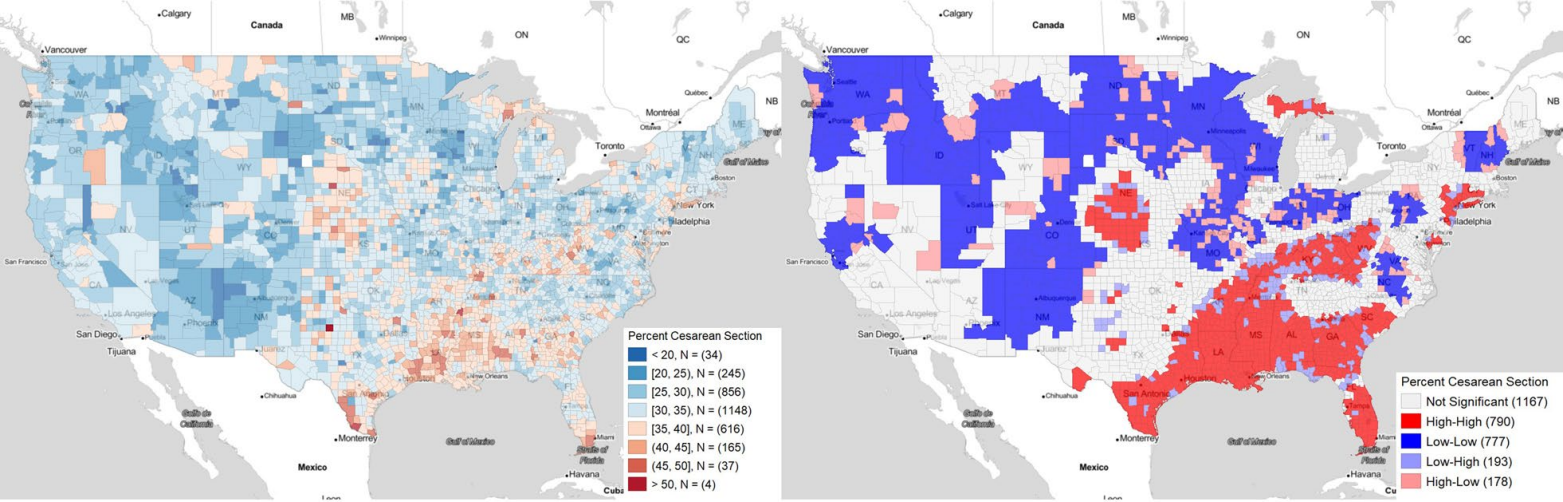
Total C-section rates at the county level: (**A**) direct county-level data (**B**) geographic clusters using Moran’s I. This figure was generated using GeoDa

Total C-section rates at the county level of the 48 continental United States are shown in Fig. 1A, B displays the geospatially clustered areas of increased and decreased C-section rates. There is a major hotspot cluster of higher C-section rates in the American South, including significant parts of eastern and southern Texas, Louisiana, Arkansas, Mississippi, Alabama, Georgia, Florida, and South Carolina. The cluster extends along the Ohio river through Kentucky and West Virginia. However, the cluster excludes most of Tennessee, North Carolina, and Virginia; the latter two in fact have decreased rates compared to nationwide averages.

There is also a separate, smaller cluster of increased C-section rates in area overlying northwestern Kansas and southern Nebraska. There are also smaller clusters of increased C-section rates in Michigan’s northern peninsula and in the New York City greater metropolitan area, which includes parts of Connecticut and New Jersey.

The American West and Midwest have coldspot clusters of decreased C-section rates. This includes large rural areas, but also major metropolitan areas such as the San Francisco Bay area, the Portland-Seattle metropolitan areas, Salt Lake City, Denver, and Minneapolis.

### Rural demographics

**Fig. 2:**
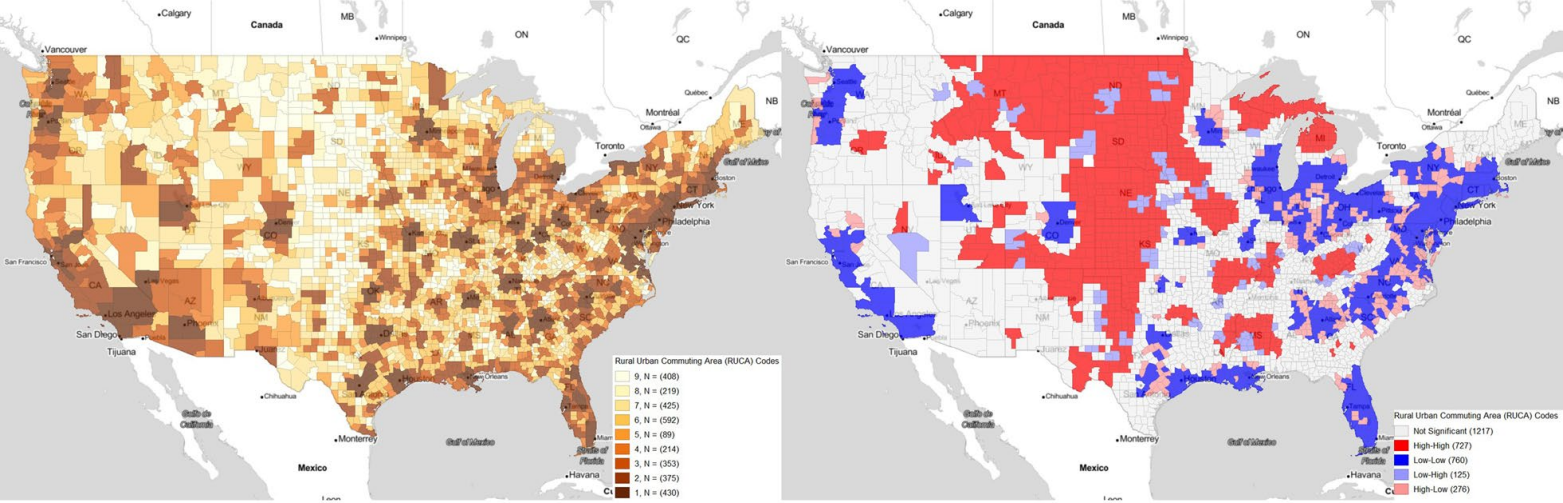
RUCA levels at the county level: (**A**) direct county-level data (**B**) geographic clusters using Moran’s I. This figure was generated using GeoDa

The RUCA code is a metric to assess how rural or urban a county is Fig. 2 displays the RUCA codes of all counties and Fig. 2B displays the geospatially clustered areas of increased or decreased urbanization. The use of geospatially clustered areas allows us to not only use absolute urbanization level, but also the relative urbanization levels.

**Fig. 3:**
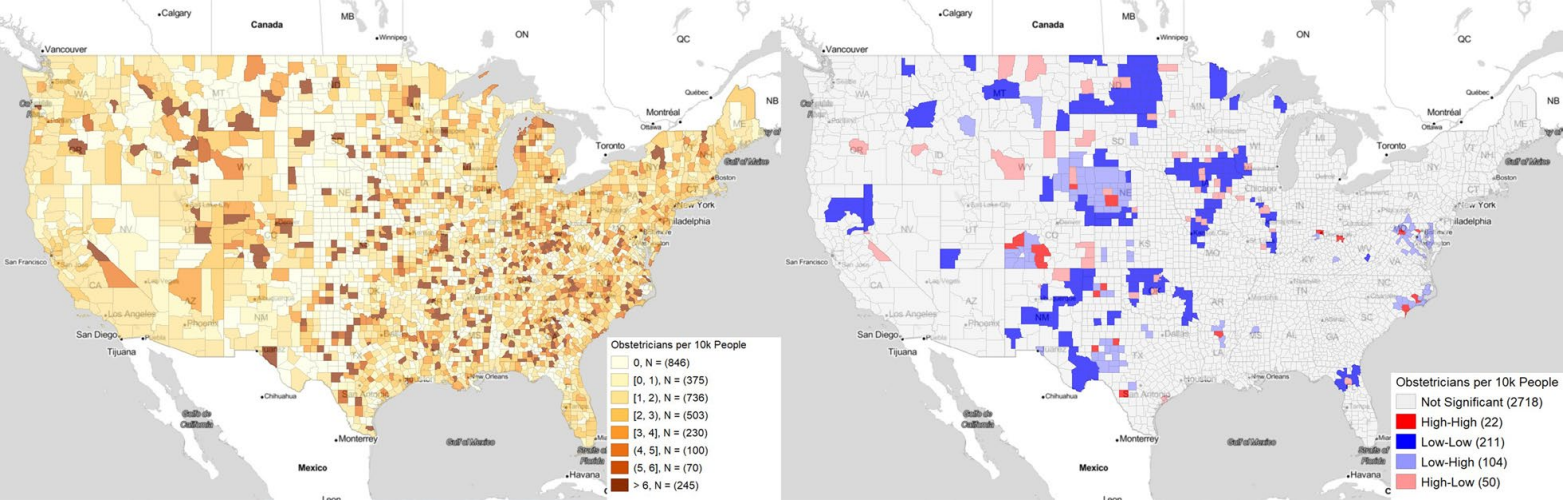
Number of practicing obstetrics-gynecology physicians per total population (**A**) direct county-level data (**B**) geographic clusters using Moran’s I. This figure was generated using GeoDa

Fig. 3A displays the number of obstetrics-gynecology physicians in a county. Fig. 3B shows this information as geospatial clusters. Most counties are non-significant, indicating a lack of sharp differences between nearby counties. There are some notable exceptions: southwestern Colorado; Union County, Louisiana; Potter County and Hutchinson County, Texas, Lincoln County, Nebraska; Scotts Bluff County, Nebraska; Frederick County and Clarke County, Virginia; Craven County, Pender County, and New Hanover County, North Carolina.

Comparing Figs. 1B and 3B, it appears regions with increased access to obstetricians were generally immune to being a low C-section rate area, although the reverse was not necessarily true. The geospatial analysis allows us to realize that those areas with more obstetricians and more C-section rates were generally surrounded by areas with fewer of both. Additionally, the overall magnitude of change between hotspots and the coldspots was small (2.2 vs 2.3 obstetricians per 10,000 people). Thus, this correlation is likely from travel from neighboring counties to higher level medical centers as opposed to a causal effect of having more obstetricians.

### Ethnic and racial factors

Additional file 2: Table S1 shows data from ANOVA analysis of C-section rates by all the demographic data collected on each county. The C-section hotspot regions generally had more individuals identifying as Black (19.6% vs 7.1%) and Hispanic (21.7% vs 13.1%), while cold spots were more likely to have more multiracial individuals (2.5% vs 3.9%) (Additional file 1: Figure S1).

Previous works have explored racial and ethnic disparities with respect to Cesarean delivery rates in depth. Okwandu et al. found that for their cohort, all other racial and ethnic groups had higher odds of Cesarean delivery compared to white women [17]. Additionally, their dataset allowed for analysis of indication, and they found that Black women had greater odds of fetal intolerance, while Hispanic and Asian women had greater odds of failure to progress [17].

Thus, the higher populations of Black and Hispanic mothers in hotspots matches expectations; the higher population of multiracial individuals in cold spots was somewhat unexpected. A weakness of the county-level dataset and methodology utilized here is the absence of indication as an available variable. As noted in previous works, these racial disparities often persist despite incorporation of variables regarding maternal and neonatal health, including variables such as fertility rate [17, 18]. In this analysis, racial categorization alone at the county level has a strong correlation with the existence and location of hotspots, supporting the findings in previous works.

## Conclusion

Our findings show that there is remarkable variation in C-section rates across the United States. These findings are not random, but also are not easily explained by any single factor. There is a strong connection between the American South and increased C-section rates. This trend is true in both rural and urban areas of the South and is true across regions of varying racial demographics. Additionally, in general, the American West and Midwest have more regions of decreased C-section rates than the Eastern United States. However, the trends have major exceptions to the above generalizations. These outlier regions, such as Michigan’s upper peninsula and the Kansas-Nebraska border, may deserve further investigation to determine the exact cause.

## Limitations

Our findings are primarily observational. We have thoroughly described the distribution of C-sections and other interesting metrics across the United States, but we can only speculate on the potential causes. Cause and effect cannot be ascertained without prospective intervention, which is not realistically feasible. Similarly, due to the nature of the dataset chosen and the granularity of the analysis at the level of individual counties, the incorporation of physiologic variables to provide meaningful data was not possible.

Geospatial analysis with Moran’s statistic also has its limitations. Our geospatial analysis is limited by our definition of neighbors, which utilized the center point of each county as opposed to accommodating for the population distribution within a county. Additionally, in this study, we only investigated the continental United States and ignored Hawaii and Alaska. As these two states are separated from the mainland, are composed of only a handful of counties, and are irregularly shaped, they do not lend themselves well to our analysis. Further work can propose alternative analysis methods for these states.

Providing an exact definition of rural or urban and condensing it down to a single metric is always circumspect. We have chosen to use standardized metrics in the literature, such as the RUCA code, for this to eliminate potential bias introduced by our work. Additionally, we estimated the access to pregnancy providers by using the number of obstetrics-gynecology physicians on an NPI database. We used this database because it is standardized and is not dependent on surveying biases. However, this does not account for those obstetrics-gynecology physicians who no longer practice obstetrics (i.e. gynecology oncologists) and does not take into account other medical professionals that can manage the delivery process (i.e. obstetrics-practicing family medicine physicians, certified nurse-midwives).

Lastly, in this paper, we restricted ourselves to total C-section rates for simplicity and due to limitations in the dataset. Further work could investigate primary C-section vs repeat low transverse C-section rates, failed TOLAC (trial of labor after C-section) vs VBAC (vaginal birth after C-section) rates, and planned vs emergency C-section rates.

## Supplementary Information

**Additional file 1: Figure S1.** Percentage of Population Identifying as Black (A) direct county-level data (B) geographic clusters using Moran’s I. This figure was generated using GeoDa.

**Additional file 2: Table S1.** Summary demographics of all counties in each C-section group identified by geospatial analysis along with the p-values of the ANOVA test and the t-test comparing high-high to low-low.

## Data Availability

The original datasets used are available publicly. The compiled, machine-readable formatting of the dataset are available from the corresponding author on request and will be made publicly available after publication of this manuscript.

## Abbreviations

ANOVA: Analysis of variations
C-section: Cesarean section
NPPES: National Plan and Provider Enumeration System
NPI: National Provider Identifier
RUCA: Rural urban commuting area
