# Supplementary figures and images for: Geospatial distribution of relative cesarean section rates within the USA
Source: BMC Res Notes. 2022 Jul 15;15:247. doi: 10.1186/s13104-022-06141-w (PMC9284873; doi:10.1186/s13104-022-06141-w)

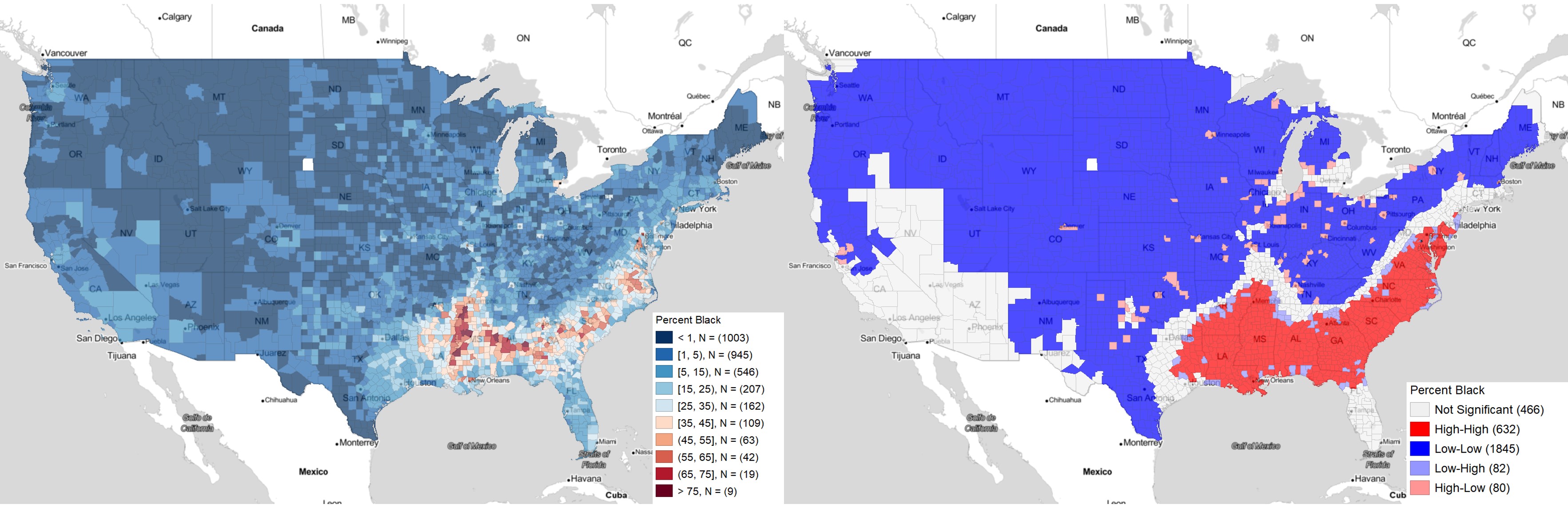

Supplement: Supplementary file 1 — Additional file 1: Figure S1. Percentage of Population Identifying as Black (A) direct county-level data (B) geographic clusters using Moran’s I. This figure was generated using GeoDa. [file 13104_2022_6141_MOESM1_ESM.jpg]
